# Rethinking the Dental Amalgam Dilemma: An Integrated Toxicological Approach

**DOI:** 10.3390/ijerph16061036

**Published:** 2019-03-22

**Authors:** Hector Jirau-Colón, Leonardo González-Parrilla, Jorge Martinez-Jiménez, Waldemar Adam, Braulio Jiménez-Velez

**Affiliations:** 1 Department of Biochemistry, School of Medicine, Medical Sciences Campus, University of Puerto Rico, Main Building B210, San Juan 00936, Puerto Rico; hector.jirau@upr.edu (H.J.-C.); leonardo.gonzalez2@upr.edu (L.G.-P.); jorge.martinez20@upr.edu (J.M.-J.); 2 Center for Environmental and Toxicological Research, San Juan 00936, Puerto Rico; 3 Department of Chemistry, Rio Piedras Campus, University of Puerto Rico, Av. Dr. José N. Gándara, San Juan 00936, Puerto Rico; wadam@chemie.uni-wuerzburg.de

**Keywords:** dental amalgam, mercury, restorative dentistry, methylmercury, oral toxicology, toxic metals

## Abstract

Mercury (Hg) has been identified as one of the most toxic nonradioactive materials known to man. Although mercury is a naturally occurring element, anthropogenic mercury is now a major worldwide concern and is an international priority toxic pollutant. It also comprises one of the primary constituents of dental amalgam fillings. Even though dental mercury amalgams have been used for almost two centuries, its safety has never been tested or proven in the United States by any regulatory agency. There has been an ongoing debate regarding the safety of its use since 1845, and many studies conclude that its use exposes patients to troublesome toxicity. In this review, we present in an objective way the danger of dental amalgam to human health based on current knowledge. This dilemma is addressed in terms of an integrated toxicological approach by focusing on four mayor issues to show how these interrelate to create the whole picture: (1) the irrefutable constant release of mercury vapor from dental amalgams which is responsible for individual chronic exposure, (2) the evidence of organic mercury formation from dental amalgam in the oral cavity, (3) the effect of mercury exposure on gene regulation in human cells which supports the intrinsic genetic susceptibility to toxicant and, finally, (4) the availability of recent epidemiological data supporting the link of dental amalgams to diseases such as Alzheimer’s and Parkinson.

## 1. Introduction

The use of mercury in tooth fillings represents some 10% of the total global mercury consumption; thus, it is the largest consumer of mercury in the world [1]: in the U.S. alone, up to 32 tons are used per year [2]. Compared to the USA, the dental use of mercury in the European Union—the second largest consumer—amounts to some 20–25%, although countries such as Norway, Denmark, and Sweden have recommend banning the use of mercury in dental amalgams [3].

The primary use of mercury amalgam in tooth fillings is to delay tooth decay. This restorative material is composed of an approximately 50% metal alloy—a mixture of silver, copper, and tin—while the other 50% consists of elemental mercury [4]. The restorative power relies on the chemical properties of the mercury to form the amalgam. Through proper guidance, the dentist mixes the powdered alloy with the liquid mercury to form amalgam putty. The pliable amalgam putty is then placed and shaped into the tooth cavity, where it is left to harden into a solid state. The benefits provided through this restorative procedure are many: it is less expensive, long-lasting, strong, and resistant, thus less likely to break than any other type of fillings [5]. Recently, however, a research group from England focusing on Alzheimer’s treatment discovered “Tidegluzib”, a novel GSK3 antagonist, which promotes natural tooth repair [6].

Mercury, the principal component in dental amalgam, derives the very well-known toxicity from its high affinity towards proteins and amino acids [7]. In vitro experiments have demonstrated that elemental mercury is ten times more toxic than lead on neurons (Pb) [8]. Tissues such as the liver, kidney, and central nervous system (CNS) are the primary targets for bioaccumulation [9,10,11]. In view of the proximity of the oral cavity to the brain, the mercury penetrates and deposits in this organ affecting the CNS. Experiments using rats have shown the immediate fate of mercury release into the brain [12,13].

Even though dental mercury amalgams have been used for more than 150 years, their safety and risks have never undergone the regulatory proof-of-safety testing that is required for other medical implants under the U.S. law. Under the 1976 Amendments to the Federal Food, Drug, and Cosmetics Act (FDA), Congress directed the FDA to assess the safety of medical and dental devices and to require premarket approval of safety for any device *“intended to be implanted in the human body”* [14]; yet, dental amalgam has been exempted by the FDA [15]. 

In 1991, the World Health Organization confirmed dental amalgams are the biggest source of mercury, exposing the people to mercury levels significantly exceeding those set for food, air and water [16]. Autopsy studies have shown dental amalgam to be the main source of mercury in human tissues, responsible for at least 60–95% of mercury deposits [8]. From the above, it should be obvious that the health hazard of mercury amalgam is a serious problem which needs urgent control.

Our point of view regarding the whole dental amalgam dilemma is based on the inherent toxicity of mercury at different levels. The bulk of the presented data focuses on the toxic effects of mercury and its derivatives. Not only is its relation to different pathological anomalies considered, as published by various groups, but also a molecular understanding of its toxicity is addressed. Our integrated approach focuses on a complete toxicity picture of the mercury constituent in dental amalgam. The various relevant toxicological factors including molecular mechanisms, gene regulation, and genetic susceptibility triggered by global gene polymorphism, are considered. 

## 2. Toxicity of Mercury and Its Compounds

The broad spectrum of effects caused by mercury compares to no other metal. Its physical properties are unique, as it exists at room temperature in the liquid state with an appreciable vapor pressure; mercury vapor is much more toxic than its liquid state [17]. Mercury exist in various oxidative states [18], namely the mercurous (Hg^+1^) and mercuric (Hg^+2^) ions, which readily react with cysteine and glutathione to form sulfides [19]. The resulting compounds are methylated by bacteria into methylmercury (MeHg) and dimethyl mercury (Me_2_Hg) and organic compounds, which, due to their greater absorption rate, are even more toxic—Me_2_Hg being the most potent neurotoxin known to date—than elemental mercury or the mercury ions [20]. It is of uttermost importance to understand the cellular damage caused by mercury and its compounds to devise appropriate regulations in the medical-implant field for the ultimate benefit of mankind.

### 2.1. Elemental Mercury

Elemental mercury (Hg^0^) is found in fish, humans, vaccine preservatives, thermometers, cosmetics, light bulbs, and other products and processes, including dental amalgams [21]. Mercury in its gaseous form (mercury vapor) is thought to come from the natural degassing of the earth’s crust, yet dental amalgam fillings are considered a significant source of this toxic gas [16,17]. The greatest concern for mercury toxicity is its gaseous state—it readily vaporizes at room temperature—because mercury vapor is odorless and invisible [7,22]. Since mercury vapor is fatal even in small amounts [7,22,23,24], lethal concentrations may be present before one realizes any symptoms (comparable to carbon monoxide). Mercury vapor is absorbed at a fast rate in the respiratory tract and consequently distributed throughout the whole body by the bloodstream [7,18]. As mercury vapor is uncharged and, therefore, highly lipid soluble, it readily passes through the blood-brain barrier and the placenta before cells oxidize it to Hg^+2^ ions [15,18,25]. The notable affinity of mercury to biomolecules, such as amino acids, proteins, purines, pyrimidines, and nucleic acids, is the reason for its toxicity, especially in the central nervous system and kidneys [7]. This interaction of mercury with these biomolecules inhibits various important enzymes, such as membrane ATPase, enzymes involved in brain pyruvate metabolism, lactate dehydrogenase, and fatty acid synthetase, causing very serious effects on the central nervous system and its metabolism [7,22]. Several studies on mercury vapor show that long-term exposures in dental workplaces (20 µg Hg per m^3^ air or higher) have detectable toxic effects on the central nervous system [16]. Concentrations of mercury greater than 0.05 mg/m^3^ for significant periods (more than 8 h) is considered unsafe by the Agency for Toxic Substances and Disease Registry (ATSDR), while a 0.2 μg/m^3^ amount is the minimum risk level from chronic mercury inhalation [24,26]. Acute and chronic exposure may induce numerous symptoms such as cough, fever, tremors, delusions, hallucinations, loss of memory, insomnia, neurocognitive disorders, personality change, and gingivitis, among others [22,26]. The daily amount of absorbed mercury estimated from atmospheric exposure in rural areas is about 32–64 ng and about 160 ng in urban areas [27]. This outdoor air exposure to elemental mercury is marginal when compared to the average estimated daily absorption through food consumption (±600 ng) or dental amalgam (3000–17,000 ng) [16].

### 2.2. Inorganic Mercury

Inorganic mercury compounds derived from mercuric ions (Hg^+2^) are formed when mercury combines with other elements, for example chlorine, oxygen, or sulfur, forming the salts mercuric chloride, mercuric oxide, and mercuric sulfide [9,12]. Also, in the human body mercury salts may be produced from elemental mercury. This happens when mercury vapor is inhaled, diffusing through the lungs into the bloodstream [7,18]. In cells, the elemental mercury is oxidized to its divalent form (Hg^+2^) by the hydrogen peroxide–catalase pathway, which occurs predominantly in the liver, lungs, erythrocytes, and brain [21]. Additionally, exogenous mercury salts are absorbed through the gastrointestinal tract, possibly via amino acid/peptide transporters and a divalent metal transporter 1 [16,25]. After absorption, these mercuric salts may produce renal failure, cardiovascular collapse, severe gastrointestinal damage, and eventually cause death [16]. Although inorganic mercury does not cause similar short-term effects as elemental mercury, long-term exposure may induce neurological disturbances and memory problems [28]. Furthermore, it has been reported that mercuric chloride (HgCl_2_) effects cell cytotoxicity, causes oxidative stress, increases *β*-amyloid secretion, and induces Tau phosphorylation in neuroblastoma cells [29]. These studies indicate that mercury exposure might play a critical role in the pathophysiological Alzheimer Disease.

While mercury vapor passes readily the blood-brain barrier, while inorganic mercury has a limited capacity to do so; however, once in the brain the toxicant is bound more strongly [18,30]. This might explain why the half-life of inorganic mercury in the brain is estimated to be 20 years, while the biological half-life is approximately 30–60 days [18]. Recent reports on human studies indicate a half-life of inorganic mercury in the brain of the order of years, contradicting older radioisotope studies which estimated half-life in the order of weeks to months [31]. Additionally, inorganic mercury may be produced from organic mercury, as experiments have shown that inorganic mercury levels within the brain correlate with organomercury administered doses [30,32]. Mercury salts have been shown to accumulate in exocrine glands, making saliva an excretion pathway [33]. A post-mortem study performed to assess mercury exposure in the human brain showed that dental amalgam increases the inorganic mercury concentrations in the brain. At time of death, a significant correlation was found between inorganic mercury in the blood and the number of surfaces filled with dental amalgam [34].

### 2.3. Organic Mercury

Organomercury refers to various organometallic compounds, specifically the extremely neurotoxic methylmercury (MeHg) and dimethylmercury (Me_2_Hg). Dimethylmercury is an extremely hazardous chemical, absorption of less than 0.1 mL produces severe and even fatal reactions [14,35,36,37,38]. According to ATSDR, the minimal risk level of dimethylmercury is 0.0003 mg/kg/day for chronic symptoms (oral exposure) [24]. The U.S. Environmental Protection Agency (US EPA) has estimated a safe daily intake for organomercury of 0.1 µg/kg body weight per day. This is based on a study in the Faroe Islands, in which development test scores for children were compared, whose mothers had been exposed to organomercury during pregnancy. A European Union scientific review in 2001 has supported this safe daily intake level [39].

It has been hypothesized that organomercury passes through the blood-brain barrier by way of neutral amino acid transporters, particularly System L [25]. Although elemental mercury affects the central nervous system, the organomercury preferentially distributes in portions of the brain that control sensorimotoric functions. The latter in turn leads to problems with coordination, equilibrium, and motoric control [10,11]. The precise molecular mechanism that produces organomercury-induced damage in the brain is still not well understood; yet, oxidative stress and lipid peroxidation represent important mechanisms in the process of neuronal death [40].

In addition to the transport of organomercury across the blood-brain barrier, such compounds may also be actively transported to a fetus through the placenta [41]. Studies have revealed that organomercury concentration in the blood of the fetus is ~2-fold higher than in the mother’s [42,43,44,45]. This poses a serious threat to the developing brain since fetuses are more sensitive to organomercury than adults [37]. Large-scale epidemiological studies have disclosed that child neurodevelopmental difficulties, including motoric function, attention, deep tendon reflexes, coordination, and visuospatial organization, are associated with pregnant women eating fish contaminated by a high organomercury levels (facilitates high in utero exposure) [37,46].

The organomercury in human tissue has been mostly attributed to food, specifically, to certain fish species. Demonstrated more than 30 years ago and now mostly overlooked is the potential capacity of oral bacteria (*Streptococcus minor*, *Streptococcus mutans*, and *Streptococcus sanguis*) to methylate elemental mercury from dental amalgams [47,48,49,50]. Consistent with this idea, patients with a higher number of mercury dental fillings exhibit larger levels of organomercury in their saliva [49,51]. Furthermore, Leistevuo reported that the levels in saliva ranged from 0 to 174 nmol/L (0–37.523 μg/L), with a mean estimate of 14.0 nmol/L (3.019 μg/L). Most (60%) salivary secretion originates from the submandibular gland [52]. The total saliva volume produced per day in five-year-old children is ~500 mL [53]; in adults the value varies from 1 to 2 L [54], a conservative estimate of saliva volume is at least 800 mL/day. Speciation analyses indicate that the mean extent of biomethylation of inorganic mercury by oral bacteria is ~2–3 μg/day [49]. A meal of about 200 g fish, which contains a high amount of organomercury (500 μg/kg), results in an uptake of approximately 100 μg organomercury; consumption of moderate levels (50 μg/kg) leads to an uptake of ~10 μg. Therefore, the weekly ingestion of one fishmeal represents in extreme cases as much as 100 μg and in moderate cases is 20 μg organomercury. In contrast, the weekly contribution of organomercury due to the biomethylation (oral bacteria) of the mercury in amalgam fillings (the average case) is equivalent to consuming moderately mercury-infested fish. Clearly, humans with dental amalgams eating mercury-containing fish are extremely endangered by mercury toxicants. Therefore, it should be evident that organomercury exposure through dental amalgams is an important and relevant health problem. More research on the toxicity of organomercury deriving from amalgam fillings is necessary to protect the health of our population.

Organomercury and mercury vapor pose a more serious threat to pregnant women and their newborns. A study in China concluded that prenatal exposure to low levels of organomercury causes smaller cerebellum fetal brain development in newborns [55]. It is advisable to perform detailed neuropsychological tests on these children after 18 months: such tests indicate latent neurological or neuropsychological deficits [56]. A Stockholm study monitored postdelivery women for 15 months: The levels of organomercury, inorganic and elemental mercury in maternal and umbilical cord blood were determined by automated alkaline solubilization/reduction and cold vapor atomic fluorescence spectrometry, whereas the total mercury in urine was determined by inductively coupled plasma mass spectrometry. Approximately 72% of the mercury in blood (*n* = 148) during early pregnancy was organomercury (median 0.94 μg/L, maximum 6.8 μg/L); it decreased during pregnancy, probably due to eating less fish. Moreover, the inorganic mercury in blood (median 0.37 μg/L, maximum 4.2 μg/L) and the total mercury in urine (median 1.6 μg/L, maximum 12 μg/L) during early pregnancy were highly correlated with the number of amalgam fillings [41]. It was not clear, however, how much of the organomercury (72%) was due to fish consumption, and how much was due to the dental amalgam. The organomercury in umbilical cord blood (median 1.4 μg/L and maximum 4.8 μg/L) was almost twice that in maternal blood, probably caused by previous exposure of the mother to mercury and the ability of the placenta to accumulate the toxicant. The concentrations of organomercury decreased during lactation, presumably due to its excretion in the mother milk. An autopsy study on deceased newborns and fetus has shown a direct correlation between dental amalgam fillings of the mother in pregnancy and the mercury levels in the body tissues of the babies or fetus [57].

## 3. Oral Exposure to Mercury Amalgam

Numerous epidemiological studies have assessed the impact of mercury exposure from oral dental amalgam. In a recent study, males with high mercury levels in hair (1 > ppm) had a 50% higher probability of having periodontitis than females with normal mercury levels (1 < ppm). The results suggest that mercury exposure, irrespective of gender, is associated to periodontitis [58]. Anaerobic bacteria from periodontal diseases produce hydrogen sulfide (H_2_S) and methyl mercaptan (CH_3_SH) are responsible for gingivitis [59]. These sulfur compounds react with the mercury amalgam to produce a black gum tissue called “amalgam tattoos”, consisting of mercuric sulfide (HgS) [60]; mercuric sulfide is extremely toxic causing oral and systemic diseases [58].

### Risks to Dental Personnel

It is worth noticing that dentists and personnel involved in amalgam restorations are at higher risk since they are exposed to more mercury vapor during a work day. An interesting study from 1992 compared a population of dentists subjected to mercury vapor to a control population with no mercury exposure. The experiment measured chronic neurobehavioral effects based on tests including motoric speed, visual scanning, visio-motoric coordination, visual memory, and verbal memory, inter alia. These performance tests showed that the population exposed daily for 5.5 years (a dose of 14 μg/m^3^, which is below the threshold limit recommended by American Conference of Governmental Industrial Hygienists) was affected significantly worse than the control subjects [61].

A recent study, performed in a dental training school on 45 students, reported mercury exposure from two sources: bound to particulate matter and from direct vapor [62]. Levels of particulate-bound mercury ranged from 0.1 to 1.2 μg/m^3^, while mercury vapor ranged from 1100 to 3300 μg/m^3^ during the clinic training. The mercury levels ranged from 0.01 to 0.02 μg/m^3^ for particle bound mercury and 13.6 to 102.7 μg/m^3^ in vapor. The mercury vapor levels were several times higher than permitted by OSHA (100 μg/m^3^). Even though there is evidence for high mercury levels in the dental working environment, regulation of the mercury levels in the clinic environments are not enforced. Clearly, personnel working in dental clinics are exposed to inhaling mercury vapor, as well as of fine amalgam particles, which comprises a potential health risk. This is due the fact that they become more exposed to mercury since they are involved in preparing amalgam, waste management, and polishing dental amalgam [63].

It had been shown that inorganic mercury induces immunosuppression by decreasing the production of thymus gland hormone (thymulin) in young mice [64]. Recently, a study in human (dental staff workers, dentists, and nurses) showed a significant increases of mercury levels in urine and blood compared to nondental personnel, and a concomitant reduction of thymulin hormone and nitric oxide in blood [65]. This study confirms the findings in humans of those previously reported with laboratory animals. Moreover, this effect was more evident in dental nurses. Yet another dental occupation study found a correlation with mercury body burden and dental amalgam vapor release. On average, urinary mercury levels were above control subjects and most of the urinary mercury was above the Health and Safety Executive health guidance value of 20 μmol/mol^−1^ creatinine [66].

## 4. Mercury Amalgam Fillings and Neurodegenerative Disorders

The constant release of mercury vapor from dental amalgam (determined by highly sensitive analytical techniques) is absorbed in blood through pulmonary airways and additionally more less amounts through the tooth pulp or gingiva [7,8,67,68]. The ability of mercury vapor to pass easily through the blood-brain barrier into the neurons and mitochondria, thereby potentially causing neurological impairment, is of significant health concern [69]. Of utmost importance is the direct relationship between the number of amalgam surfaces and mercury accumulation, as evidenced in brain tissue of cadavers [16,50,70,71,72,73]. As the brain is a nearby target for mercury uptake, its bioaccumulation in the brain may be the cause for various neurological diseases such as Parkinson and Alzheimer’s Diseases or even Amyotrophic Lateral Sclerosis [74]. Even if there were low mercury levels in the brain, genetically susceptible individuals, particularly, may be at higher risk [7,9,18]. In a study using brain tissue from autopsies on 32 individuals—10 with dental amalgam fillings and 22 without—the mercury deposition was determined in the parietal lobe. It was found that 60% of the subjects with amalgam fillings had considerably higher levels of mercury compared to only 36% of the amalgam-free group [75]. Nevertheless, no correlation was found between the mercury levels in the brain of the subjects with dental (mercury level of 0.97 ± 0.83 µg/g) or without dental amalgam (mercury level of 1.06 ± 0.57 µg/g); however, these results are not reliable because some patients were included whose teeth had been removed but had dental amalgam in earlier times. The level of total mercury in the brain was determined to be in the µg/g range (see above), while the level of organomercury was reported in the range of 4 to 5 ng/g, indicating that organomercury is an order of magnitude lower [34].

For all these years the lack of convincing epidemiological investigations which demonstrate possible links between dental amalgams and neurological disorders have been strong arguments against removing dental amalgams. New epidemiological studies are starting to emerge providing stronger evidence favoring a connection of dental amalgams with some neurological diseases. While little if any convincing evidence had been reported in most studies, a New Zealand investigation claimed a link between amalgam exposure and multiple sclerosis [75]. Extensive epidemiological work in Taiwan (using over 200,000 subjects) reported a higher risk of Alzheimer disease for individuals (age 65 and over) with dental amalgams compared to the no-amalgam control. The data showed that Individuals exposed to amalgam fillings had higher risk of Alzheimer’ s disease (odds ratio, OR = 1.105, 95 % confidence interval, CI = 1.025–1.190) than their nonexposed counterparts. The ‘odds ratio’ for Alzheimer’s disease was 1.07 (95 % CI = 0.962–1.196) in men and 1.132 (95 % CI = 1.022–1.254) in women [76]. For the first time we have been alarmed about an association between amalgam exposure from dental fillings and gender. Moreover, a survey (also from Taiwan) on more than 20,000 individuals revealed the impact of dental amalgam on the development of Parkinson’s disease. Patients with amalgam fillings had a significantly higher risk of PD after adjusted hazard ratio HR = 1.583, 95% confidence interval (CI) = 1.122 ± 2.234, *p* = 0.0089) than those who did not [77]. Clearly, these recent data are slowly emerging, and some are presenting a direct links between dental amalgam, Alzheimer’s, and Parkinson’s diseases.

## 5. Genetic Susceptibility and Mercury Exposure

A genetic predisposition, also referred as genetic susceptibility, represents a likely increase of developing a particular disease based on a person’s genetics. These alterations correlate with the development of distinct pathologies, yet are usually not considered a direct cause. Thus, it is important that these individual genetic differences be taken into consideration when dealing with the toxic mercury effects. Polymorphic human genes that mediate the toxicokinetics of mercury influence its bioaccumulation and toxicity. A study performed in an Amazon community (Brazil) exposed to organomercury assessed how polymorphisms are associated with organomercury detoxification. Given that the biotransformation of organomercury uses mainly glutathione (GSH) in the bile, mediated by glutathione transferase [78], polymorphisms in the GSTM1 and GSTT1 genes alter the levels of this neurotoxin. Their study displayed individual variations of the mercury levels in blood and hair of 71 men and 73 women. The genetic variations suggested that the GSTT1 gene plays an important role in mercury metabolism. In this population the reduced enzyme activity also decreased mercury excretion via Hg-GSH conjugation, which raised mercury retention in the body. No significant correlation was noted between GSH levels and GST polymorphisms, suggesting that other genetic factors may influence this phenomenon [79,80]. Moreover, there is evidence for other potentially harmful mercury effects such as DNA methylation, particularly at the TCEANC2 region (a transcription elongation factor). A shift in methylation within blood cells constitutes another mercury effect and manifests inability of detoxification [81].

In order to evaluate the effect of mercury exposure on gene regulation in the liver, a recent study was performed using Affymetrix oligonucleotide microarray with probe sets complementary to more than 20,000 genes. The incentive was to determine whether patterns of gene expressions differ between controls and mercury-treated (1–3 μg/mL) cells [82]. The results from a cluster analysis identified 2211 affected genes. Most of these genes were downregulated, while forty-three were significantly overexpressed. The transforming growth factor beta (TGF-*β*) superfamily of cytokines was overexpressed (associated with regulating the cell cycle essentially for maintenance of normal immunological homeostasis and lymphocyte proliferation). Many of the genes are categorized as control and regulatory genes for metabolic pathways involving the cell cycle (cyclin-dependent kinases), apoptosis, cytokine expression, Na^+^/K^+^ ATPase, stress responses, G-protein signal transduction, transcription factors, DNA repair, as well as metal-regulatory transcription factor 1, MTF1, HGNC, and ATP-binding cassette (ABC transporters), among many others. Significant alterations in these specific genes provide new directions for deeper mechanistic investigations. These would lead to a better understanding of the molecular basis of mercury-induced toxicity and human diseases that may result from disturbances in the immune system.

The ABC transporter genes have been implicated with organomercury toxicity as a cause for neurodevelopmental defects, for which polymorphisms is responsible. In particular, the SNPs in the ABC transporter maternal genes have been implicated with organomercury concentrations in hair during pregnancy, which affects child development. Of these genes, seven were associated with concentrations of mercury in maternal hair, of which one SNP is highly effective in neurodevelopment of the child. Implications for these doses of organomercury in the development of the child is still to be evaluated and further investigated, yet variation in ABC transporters genes are related to maternal mercury concentrations [83]. To stress, correlation does not imply causation; thus, these ABC transporter genes need further research of the organomercury metabolism in genetically predisposed individuals.

Adding to these recent studies a genetic association of single nucleotide polymorphism was performed on 308 participants from an ADA (2012) annual meeting [84]. Single nucleotide polymorphisms (88 SNPs in classes relevant to Hg toxicokinetics) were evaluated in samples of hair, blood, and urine as possible Hg exposure biomarkers. A total of 38 SNPs were suggested as candidates to influence Hg biomarker levels. These SNPs were associated with glutathione metabolism, selenoproteins, metallothioneins, and xenobiotic transporters. Among their findings were the SNPs of rs732774 (BDNF) and rs1061472 (ATP7B) associated with lower hair Hg, and GCLC SNP rs138528239, which is associated with lower bHg concentrations overall. This work expands on the list of previously discussed genes. Moreover, it is important to mention that mercury toxicity in neurons is mediated by calcium in addition to the ones mentioned above [85].

A classic study was performed at the University of Washington where the susceptibility to mercury exposure was investigated and how it varies with the individual genetics. The polymorphisms of various children genes, particularly metallothionein, were examined. This family of proteins participate in the distribution and excretion of mercury and other toxic metals; moreover, MT1 and MT2 gene isoforms are involved in the mercury dispersal and storage of the central nervous system [11]. The distribution and storage of mercury will depend on the differences of the proteins and SNPs in relevant genes including those involved in glutathione metabolism, selenoproteins, metallothioneins, and xenobiotic transporters. The same study evaluated other proteins, which are crucial in mercury metabolism, namely catechol-O-methyltransferase (COMTregulates catecholamine neurotransmitters), the tryptophan 2,3-dioxygenase (TDO2 is involved in the rate-limiting step in the catabolism of tryptophan), and GRIN2A-GRIN2B (glutamatergic receptors, which mediate central nervous system excitatory neurotransmission). Neurodegenerative disorders have also been associated with mercury exposure and regulation of gene expression. Some of these disorders are Parkinson’s disease, myalgic encephalopathies, epilepsy, Alzheimer’s disease, and others. Studies have shown a correlation in psychiatric disorders—mainly anxiety and panics fits—due to chronic mercury toxicity [86].

Mercury toxicity, for example, in neurological health, has been widely studied in regard to the ability of the toxic metal to surpass freely the blood–brain barrier. One of the most explored cases is Alzheimer’s disease, as previously discussed. A report points out that polymorphisms in the lipoproteins APO-E2, APO-E3, and APO-E4 are related to an increased risk for Alzheimer’s disease. A correlation has been established between sulfhydryl (HS) groups on the surface of the APO-E protein (binds Hg^2+^ ions) and the removal of mercury from the brain. The increased risk of acquiring the Alzheimer’s disease may be up to 80% greater if the APO-E4 form predominates over the APO-E2 form [60]. Since these housekeeping APO-E proteins are involved in removing toxic metals (including mercury), as well as oxidized lipids, this condition may exacerbate in genetically sensitive people [85]. The levels of mercury in the brain of Alzheimer’s-affected subjects need to be evaluated in accordance to genetic susceptibility of previously discussed genes and the pathological conditions.

## 6. Summary

Enough data have been presently assembled to be concerned with the mercury health problem. The mayor sources of mercury in humans are dental amalgams and the food chain. The constant release of mercury and its presence in saliva, as well as the added consumption of contaminated fish and seafood products, constitute a serious and exacerbated burden in humans. However, much of the commercial fish are now being grown in fish hatcheries and the restrictions of release of mercury into the environment are enforced in many countries, which should lead to a reduction in mercury body burden in the future. Although much of the detrimental mercury is eliminated, part of it is accumulated and biotransformed into organomercury compounds. These find their way into the brain, where they may persist in the order of years. Due to such acute or chronic exposure, many pathological conditions have been ascribed to mercury toxicity: immunosuppression, neurological disorders, cardiovascular diseases, hormonal imbalance, and gingivitis, to mention a few of the more serious ones. Consequently, the development of the pathological conditions associated with mercury exposure constitutes a serious health burden, which adds constraints and limits lifespan. Indeed, recent studies have revealed an association of dental amalgams with Alzheimer and Parkinson disease. Many genes (GCLC, MT1M, MT4, ATP7B, and BDNF, currently used as biomarkers) respond to mercury exposure, which either enhances mercury excretion or accumulation. Therefore, relevant individual polymorphism in mercury-responsive genes can alter its availability, bioaccumulation in specific tissues and, hence, its toxicity.
